# Plant CDKs—Driving the Cell Cycle through Climate Change

**DOI:** 10.3390/plants10091804

**Published:** 2021-08-30

**Authors:** Aline Köhn Carneiro, Patrícia da Fonseca Montessoro, Adriana Flores Fusaro, Bruna Gino Araújo, Adriana Silva Hemerly

**Affiliations:** Laboratório de Biologia Molecular de Plantas, Instituto de Bioquímica Médica, Universidade Federal do Rio de Janeiro, Cidade Universitária, Rio de Janeiro 21941-902, Brazil; alinekohncarneiro@gmail.com (A.K.C.); patriciamontessoro@gmail.com (P.d.F.M.); adriana.fusaro1@gmail.com (A.F.F.); brunagino95@gmail.com (B.G.A.)

**Keywords:** climate change, cell cycle regulators, cyclin-dependent kinases, CDK, stress adaptation, plant plasticity

## Abstract

In a growing population, producing enough food has become a challenge in the face of the dramatic increase in climate change. Plants, during their evolution as sessile organisms, developed countless mechanisms to better adapt to the environment and its fluctuations. One important way is through the plasticity of their body and their forms, which are modulated during plant growth by accurate control of cell divisions. A family of serine/threonine kinases called cyclin-dependent kinases (CDK) is a key regulator of cell divisions by controlling cell cycle progression. In this review, we compile information on the primary response of plants in the regulation of the cell cycle in response to environmental stresses and show how the cell cycle proteins (mainly the cyclin-dependent kinases) involved in this regulation can act as components of environmental response signaling cascades, triggering adaptive responses to drive the cycle through climate fluctuations. Understanding the roles of CDKs and their regulators in the face of adversity may be crucial to meeting the challenge of increasing agricultural productivity in a new climate.

## 1. Introduction

Although the world’s climate dynamics follow a similar pattern over the years, it is known that small variations in temperature and rainfall on Earth cause a great impact on all beings that live here, whether they are microorganisms, plants, or animals [1]. A major concern in this century is the intensification of climate fluctuations [1]. This change exacerbates the characteristics of the climate during the seasons, causing long periods of drought, extremely high temperatures, or the opposite: super-low temperatures, increased UV radiation, and even increased salinity [2]. As plants are sessile organisms, they have to directly deal with these climatic variations, which are not always overcome, causing large losses in agriculture and increasing food insecurity in the world population [3]. Using historical climate simulations to estimate crop yield losses in West Africa, it was seen that in the simulated decade between 2000 and 2009, the climate was 1 °C warmer, and this reflected in productivity losses of 10–20% for millet (*Pennisetum glaucum*) and 5–15% for sorghum (*Sorghum bicolor*) [4]. Among the environmental changes that affect crop yield, the increasing temperature is the most problematic one [5]. It is estimated that global losses in yield would be in the order of 3.1% for soybean, 3.2% for rice, 6.0% for wheat, and 7.4% for corn for each Celsius degree increase in global mean temperature (assuming that there is neither genetic improvement nor crop adaptation) [6].

Plants respond to the environment through the plasticity of their body and their forms, which are modulated during their growth. Phenotypic plasticity can be translated into the ability that plants of the same genotype have to respond differently to better adapt to adverse conditions. It is a very important concept when dealing with the effects of climate change on plants [7]. The term plasticity is closely linked to the term stability; the latter is widely used by plant breeders to refer to the greater or lesser ability of genotypes to adapt to climatic fluctuations, over agricultural years, within a given location [8]. Adapting to new conditions means changing their morphology and physiology. The morphology is mainly modulated through the activity at the self-perpetuating meristems that can be shaped by developmental and environmental signals, imposing an accurate balance of cell division and cell differentiation to end with the correct form [9]. In organogenesis, the number of cells in a tissue is controlled by the mitotic cell cycle, while the increase in cytoplasmic volume (cell expansion) can be associated with an alternative cell cycle, called endoreduplication; therefore, changes at the molecular level of cell cycle controls translate into phenotypic changes [10]. The plant growth, being tightly connected with the environment, has critical consequences for agriculture, as, in general, plants decrease their cell division rates to better adapt, leading to a reduction in yield [11].

The cell cycle is a highly regulated process to ensure the fidelity of the replication of its genetic material, followed by its division between two daughter cells [12]. Cell cycle progression is driven by a family of serine/threonine kinases called cyclin-dependent kinases (CDK), which are activated by their cyclin partner, allowing the passage of the G1/S and G2/M phases [12]. Since the first CDKs were described in plants [13], they have been extensively studied, and there is ample knowledge about their role as a key element of the basic machinery regulating the cell cycle [14,15,16]. However, cell division regulators acting during unfavorable plant developmental conditions are still being unveiled. This work aims to gather data in the literature on the performance of the cyclin-dependent kinases (and some of their regulators) in the face of various environmental setbacks to elucidate the important role of the cell cycle as a member of the environmental response signaling cascade. Finally, in this scenario, we propose that the protein kinase CDK has a dual role as a key regulator of the cell cycle progression: (a) to accurate balance cell division and cell differentiation, to end with the correct plant form (developmental controls) and; (b) as well as being part of intracellular signaling cascades that respond to several environmental stresses, by modulating cell division rates that are key for environmental plasticity and adaptation. For these reasons, understanding the roles of CDKs and their regulators in the face of adversity may be crucial to meeting the challenge of increasing agricultural productivity in a new climate.

## 2. A Brief Description of Plant Cyclin-Dependent Kinases and Their Role in Plant Growth

**Figure 1:**
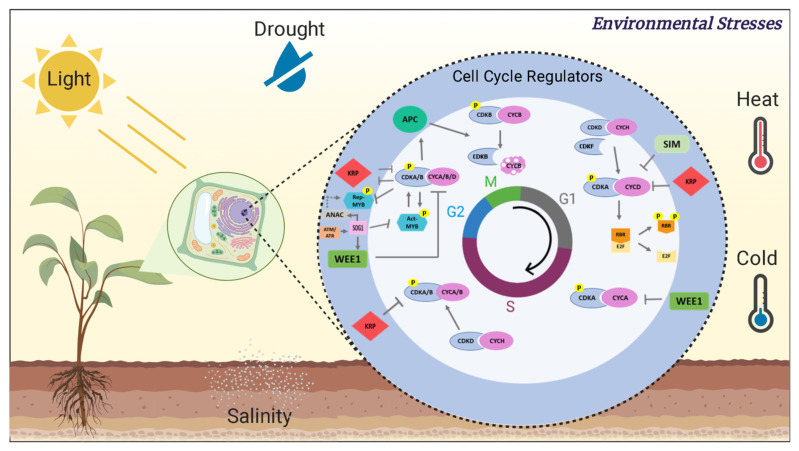
The influence of the environment on cell cycle regulation. The proliferation of cells in plants is composed of two successive steps: interphase and mitosis. At the end of G1, CYCD-CDKA phosphorylates the RBR protein, thus releasing the E2F and DP heterodimer, promoting transcription of key genes for G1/S transition. CYCDs are active in both G1/S and G2/M. During the cycle, the CDK-activating kinase (CAK) proteins, CDKD and CDKF, act by phosphorylating and activating CDKA/B. CDKF has independent activity, unlike what occurs with CDKD, which requires interaction with cyclin H. In G2, CDKA/B interact with CYCA/B/D and phosphorylate and activate Act-MYB, which in turn induces the expression of G2/M specific genes, including mitotic cyclins that interact with CDKA/B. In contrast, CDKA/B–CYCA/B/D phosphorylate and destabilize Rep-MYBs to prevent their binding to the MSA element, which is important for the G2/M transition. Under unfavorable environmental conditions, CDK inhibitors such as WEE, SIM, and KRP sense external signals and inhibit CDK activity to arrest the cell cycle. DNA damage is sensed by ATM/ATR, which phosphorylate and activate SOG1 to trigger DDR. SOG1 induces the expression of ANAC044/085 and CKIs and indirectly represses Act-MYB expression. ANAC044/085 stabilize Rep-MYBs, which in turn repress the expression of G2/M-specific genes arresting the cycle at G2. During mitosis, CDKA/B phosphorylate and activate APC/CCDC20, promoting the destruction of CYCB and consequently the inactivation of CDKB to allow mitotic exit. Abbreviations: ACT-MYB—activator-type MYB3R; REP-MYB—repressor-type MYB3R; SOG1—suppressor of gamma response 1; ATM—ataxia telangiectasia mutated; ATR—ATM- and RAD3-related; DDR—DNA damage response; CDK—cyclin-dependent kinase; CYC—cyclin; APC/C—anaphase-promoting complex/cyclosome; SIM—siamese; CKI—CDK inhibitor; NAC—domain transcription factors; KRP—ICK/KIP-related protein. Dotted arrow—indirect mechanism; continuous arrow—induction; block arrow—repression. Created with BioRender.com.

As well as its major-related proteins, the cell cycle is highly conserved among eukaryotes, allowing the study of the progression of the cell cycle using different organisms [12]. Although each organism has its peculiarities concerning these actors, cell cycle progression is driven by a class of serine/threonine heterodimeric kinases common to all eukaryotes. These kinases are made up of two subunits: an inactive catalytic subunit and a regulatory-associated cyclin. The cell cycle dynamics require the orderly participation of CDK/cyclin complexes for phase progression; therefore, CDKs and cyclins must act rhythmically and together [15]. The kinase phosphorylating activity occurs in serine or threonine residues of the target protein, within a recognition motif formed by the amino acids Ser/Thr-Pro-n-Arg/Lys, where n represents any amino acid. Besides CDK levels being constant, the kinase activity is cyclical, i.e., the presence and association of the cyclins in the CDK-cyclin complex is the main regulator of kinase activity, which is also regulated by CDK phosphorylation/dephosphorylation, interaction with inhibitory proteins, and targeted proteolysis of the cyclins [15] (Figure 1).

The first identified CDK–cyclin partners have a well-known role in G1/S and G2/M checkpoints, despite not all of the known kinases being involved in the control of cell cycle progression [12]. Arabidopsis (*Arabidopsis thaliana*) has more than 70 cell cycle regulators, including CDKs and cyclins [11]. Its genome has eight groups of genes encoding CDKs [17], and over 50 genes that encode different types of cyclins, classified in the groups A, B, C, D, H, L, T, U, SDS, and CYCJ18 [12]. Remarkably, compared to animals, plants have many more members in the CDK and cyclin families. Some of these members are unique and can only be found in plants [18]. This evolution in the number of plant CDKs and cyclins means that different complexes can phosphorylate a wide range of substrates in different phases and different tissues, establishing various levels of regulation [11]. This diversity of regulation might allow better performance for plants due to their sessile lifestyle and their intrinsic need to survive to the different setbacks to which they are exposed [12]. Table 1 summarizes the phenotypes of some of the CDK and cyclin mutants.

### 2.1. The Catalytic Subunity of Cyclin-Dependent Kinases (CDK)

The conserved cyclin-binding domain PSTAIRE was described for the first identified CDK in eukaryotes [12], but variations in the sequence of this motif have been identified in other members of the family [12]. These variations (which can occur in one or more of the seven amino acids) are used to classify these kinases into subgroups. All eukaryotes have at least one member of the PSTAIRE CDK [19]. Plants have eight types of CDKs: seven are classified by letters, from A to G, according to their similarity to kinases already described in animals, and the eighth is a CDK-like kinase (CKL) [11,20]. A-type (CDKA) and B-type CDK (CDKB) play an essential role in the plant cell cycle regulation [12]. Table 2 shows the members of the CDK family described in Arabidopsis, the binding motif to cyclins, and its homologs in mammals.

The plant CDKA is a homolog to yeast CDC2/CDC28 and mammalian CDK1 and CDK2, having the PSTAIRE domain [13]. CDKA is well known for its role in the G1/S and G2/M mitotic transitions. CDKA involvement in the G1/M transition has already been demonstrated in two mechanisms. The phosphorylation target of the CDKA/CYCD complex is the transcriptional repressor E2Fc/DP/RBR, which is marked to be recognized and ubiquitinated by SCF E3-ubiquitin ligase and further destroyed by the 26S proteasome. Another target of CDKA/CYCD is the retinoblastoma-related protein (RBR) which releases the transcriptional activity of E2Fa/b/DP when phosphorylated [16]. In the G2/M transition, there is the activity of both CDKA and CDKB that can associate with CYCD, CYCA, and CYCB. These CDK/CYC complexes have a vast amount of proteins as phosphorylation targets [21], and once this complex becomes active, the G2/M transition is licensed [12]. The use of dominant-negative mutants for CDC2a (CDKA) showed that morphogenesis and developmental timing were not affected in tobacco (*Nicotiana tabacum*) mutants with low kinase activity [22]. It was the first molecular evidence that, in plants, the developmental controls that define their shape could act independently of cell division rates, suggesting flexibility in these mechanisms that regulate the balance between cell division and expansion [22]. *cdka-1* mutants in Arabidopsis demonstrated a discontinuous epidermis with gaps between the anticlinal walls of neighboring floor cells, indicating a relationship with cell expansion processes [23]. However, the dominant *CDKA;1* mutant reduced cell division in seeds, resulting in defective embryogenesis [24], suggesting that cell division events are essential for embryo patterning.

There is also a role of CDKA in embryonic development, suggested through evidence of male gametophyte lethality, demonstrating the essentiality of cell division in male gametogenesis [25]. Transgenic tomato plants overexpressing CDKA;1 developed fruits with a reduced number of seeds and mucilage comparable to the wild type [26]. CDKA has also been recognized for its role in meiosis. Low CDKA activity in the *cdka;1^DBD^* Arabidopsis mutant led to a progressive loss of meiotic cross-over class I [27]. The ASYNAPTIC 1 (ASY1) protein, a target of CDKA phosphorylation, is crucial for chromosomal axis formation in Arabidopsis, being an important regulator of chromosomal synapse and bivalent formation [28]. Finally, mutant maize plants for the *CDKA;1* gene, which were directed to specifically regulate endoreduplication, underwent a modification, resulting in the loss of phosphorylation activity. Lower levels of endoreduplication did not affect cell size and only minimally reduced starch and storage protein accumulation [29].

Type-B cyclin-dependent kinases belong to a particular type of CDK class only found in plants. They are the first non-PSTAIRE kinase described and have four members distributed in two groups B1 and B2, CDKB1;1/CDKB1;2, and CDKB2;1/CDKB2;2 respectively [18,30]. CDKB1 has a PPTALRE motif and CDKB2 has a PPTTLRE motif, and these cyclin-binding motifs differ from the original PSTAIRE motif by the substitution of two or three amino acids, respectively. Type-B CDKs are specifically involved in regulating the G2/M phase transition [18]. CDKB2;1 and CDKB2;2 are fundamental for cell cycle progression and for the organization of SAM. cdkb2 mutants have an interruption of the functional CDKB2 gene that causes severe defects in the meristem [31]. A negative feedback loop between CDKA and CDKB has been proposed in a study using *Chlamydomonas reinhardtii*. In this regulatory loop, CDKA activated CDKB-cyclin B and, on the other hand, CDKB-CYCB allowed the entry of the cycle into the M phase, inactivating CDKA-CYCA [32].

As stated earlier, CDK must be complexed with its partner cyclin to become active. However, that is not all. For the substrate to bind to the enzyme’s catalytic site, the flexible domain called T-loop must be phosphorylated at its threonine residue. This phosphorylation is completed by a CDK-activating kinase (CAK), which itself is a member of the CDK family [33]. Plants have two described CAK—CDKD and CDKF [33]. CDKD has three known members, CDKD;1, CDKD;2 and CDKD;3, which form an active complex with cyclin H and are homologous to CDK7-CYCH in vertebrates [33]. The CAKs will be discussed further down in this review.

CDKC, CDKE, CDKG, and CKL classes are the most recently described and least explored, with their function in the cell cycle remaining to be determined. CDKC [34,35] is the plant ortholog for CDK9 in vertebrates, and its phosphorylation target is the C-terminal domain (CTD) of RNA polymerase II, having a described role in DNA transcription [5]. Furthermore, CDKC already had its role described in plant immunity [36]. Microbe-Associated Molecular Patterns (MAMPs) triggered phosphorylation of the RNA polymerase II CTD by CDKC in Arabidopsis, which itself is phosphorylated and activated by a MAPK cascade. CPL3 (CTD phosphatase-like 3), which is a negative regulator of the immune gene expression, dephosphorylates the CTD of RNA polymerase II. Therefore, CDKC modulates the phosphorylation state of the transcription machinery, showing its role in the host’s immune response [36].

CDKE in plants is the corresponding homolog to CDK8 in animals. HUA ENHANCER 3 (HEN3) encodes CDKE in Arabidopsis and plays a role in cell differentiation, being required in floral meristems to specify the identities of plant sex organs (stamens and carpels) [37]. CDKE also seems to have a central role in transcription regulation during stress periods [38]; it is a component module of the mediator complex, being able to both positively and negatively regulate the transcription depending on the stress it is undergoing [39].

CDKG is a subfamily of CDKs that is related to CDK10 and CDK11 in mammals [40]. CDKG has a recognized role in meiosis by acting on the pairing of homologous chromosomes and synapse completion, suggesting a mechanism to explain thermal sensitivity in male meiocytes; besides, CDKG acts in mRNA processing, particularly in alternative splicing [41]. The cdkg;2 homozygous knockout mutant (SALK_090262) showed delayed shoot regeneration compared to the Arabidopsis wild type, dwarfism, and did not develop roots, suggesting that CDKG;2 affects morphogenic responses in culture, probably in the root meristem formation [42].

The last described group of kinases is called cyclin-dependent kinases-like. In Arabidopsis, they range from CKL1 to CKL15, where the residues of the cyclin-binding motif (V, I, L) (K, R) FMAREI change according to the type of CKL involved [43]. CKL is involved in the phosphorylation of a wide range of cell cycle regulators [20]. Boden et al. [44] found that *PAIRING HOMOEOLOGOUS 1 (Ph1)* gene from wheat (*Triticum aestivum*), which acts by repressing interactions between genetically similar chromosomes during meiosis, had a genetic identity with CDK-like proteins, showing the regulatory character of the cell cycle kinases of this type.

### 2.2. Cyclins—The Activating Subunit

Cyclins play a prominent role in development, together with CDKs. Relying on a large number of mitotic cyclins, plants have a fine and rigorous adjustment in the cell cycle [12]. The key cyclins that directly control the cycle are the CYCA and CYCB, which are mainly expressed in the G2/M transition, and CYCD, which regulates the G1/S progression [45].

*CYCA1;2*, called *TARDY ASYNCHRONOUS MEIOSIS (TAM)* in Arabidopsis, is active in the meiotic cell cycle. *cyca1;2* loss-of-function mutants failed to enter meiosis II and showed an increase in the size of the nucleus in trichomes and guard cells [46]. Surprisingly, overexpression also led to a similar nucleus phenotype that could be explained through a possible post-transcriptional regulation [47]. cyca2 triple mutant plants showed defects in cell cycle progression and in newly formed guard cells [48]. *CYCA1* and *CYCA2* are expressed from S to M phase, unlike *CYCA3*, which is induced in the G1/S phase and remains until G2 [40]. The antisense expression of *CYCA3;2* in tobacco impaired the formation of callus in explants, in addition to inducing malformation in the embryo [49].

The first Arabidopsis cyclins identified were class B cyclins [50]. Important for the recognition of DNA damage, CYCB1 also acts in plant development, blocking mitotic activity. Different subgroups of CYCB mutant plants, such as *cycb1;1*, *cycb1;2*, *cycb1;3*, *cycb1;4*, *cycb1;1/1;2*, *cycb1;1/1;3*, *cycb1;1/1;4*, *cycb1;2/1;4* and *cycb1;3/1;4*, did not show altered root phenotype; however, they were hypersensitive to cisplatin, a drug that causes DNA damage [51]. *CYCB2;2* overexpression in rice resulted in an increase in root length [52]. 

D-type cyclins in Arabidopsis are associated with proliferating tissues. Overexpression of *CYCD3;1* altered leaf architecture in transgenic plants through hyperproliferation of leaf cells. The increased expression of *CYCD3;1* decreased the proportion of cells in the G1 phase of the cell cycle due to the increased activity of the kinase that interacts with it [53]. This cyclin plays an important role in the stem meristem. *cycd3*-suppressed mutants showed an inability to initiate shoots from calli, in addition to having a role in the development of lateral organs [54]. Additionally, *cycd3*-suppressed mutants showed a reduction in vascular cambium cross-sectional area, and when combined with ant-9 suppression, Arabidopsis double mutants showed an even greater reduction, thus indicating the interaction of the two in secondary cambium thickening [55]. Tobacco plants with increased *AtCYCD2* gene expression showed an acceleration in all developmental stages [56]. Furthermore, CYCD4 is associated with the formation of stomata precursor cells in the hypocotyl [57].

Given the phenotypes showed by loss-of-expression and overexpression mutants, it is clear that cyclins play a fundamental role in the cell cycle machinery by activating CDK kinase activity. Thus, failures in the CDK-CYC pathway affect development and impair adaptation to dynamic environmental conditions [11].

## 3. Regulators of CDK Activity

Plant growth depends on the correct coordination of cell division [58]. CDKs are at the center of these controls, acting in response to environmental signals and inner developmental stages [14]. CDK-cyclin activities can be positively or negatively regulated, such as by the binding or proteolytic degradation of cyclins (CYCs), phosphorylation or dephosphorylation, and also through the association with CDK inhibitors (CKIs) [59]. In Table 3, there are some examples of mutant phenotypes of CDK regulators.

### 3.1. Inhibitors of CDK Activity

CDK inhibitors (CKIs) may associate with the CDK/cyclin complex and inhibit the kinase activity [60]. Two CKI families are especially found in land plants with important roles in cell cycle regulation: the INTERACTOR/INHIBITOR OF CDK/KIP-RELATED PROTEIns (ICK/KRPs) and the SIAMESE-RELATED PROTEINS (SMRs). These CKI families are abundant in plants, possibly coordinating the crucial processes of development, interacting with environmental and growth signals [61,62,63]. 

Arabidopsis plants have seven ICK/KRPs that interact more commonly with CDKA;1 and different CYCD types, such as CYCD3 and CYCD4 [63,64]. Moreover, the ICK/KRP genes are expressed in most tissues to different levels, correlated with a decrease in CDK activity. Members of the ICK/KRP family may be regulated by some hormones and environmental factors [61,65]. 

ICK1/KRP1 was the first inhibitor described in plants, being induced under inhibitory conditions to plant cell division, such as treatment with Abscisic Acid (ABA) and low temperatures [66]. Overexpression of ICK1/KRP1 in Arabidopsis reduced CDKA and CYCD-type activity and decreased cell numbers, leading to inhibition of growth in vegetative and reproductive organs, with leaves and flowers presenting a serrated phenotype [64]. Similarly, the overexpression of other ICKs/KRP members in Arabidopsis, such as ICK2/KRP2 and ICK4/KRP4, promoted the same distinct phenotype of narrower, smaller, and serrated leaves due to reduction of CDK activity; while studies with multiple ICK/KRP mutants demonstrated an increase of CDK activity and growth improvement [63,67,68]. Plants overexpressing ICK2/KRP2 also showed an altered root system and modifications in reproductive organs size and morphology, besides infertility [69]. Kinematics analysis indicated an increase of the cell cycle duration in KRP2-overexpressing plants due to cell division inhibition, but curiously, the timing of differentiation in these plants remained unchanged [63]. Similar to CDKA dominant-negative mutants, plants overexpressing ICK/KRP members demonstrated the enlargement of cells as compensation to the decreased cell numbers. In addition, the cell enlargement occurred independently of endoreduplication since KRP1 and KRP2 overexpression inhibits the endoreduplication cycle [63,64].

The SIAMESE-RELATED PROTEINS (SMRs) are also abundant in plants and have particular roles in the cell cycle and growth coordination. *SIM* was the first identified *SMR* family member, being highly conserved in all land plant genomes [62,70]. A considerable number of 17 *SMR* genes are already described in Arabidopsis and are typical of angiosperm genomes [70]. Arabidopsis plants overexpressing *SIM* showed similar phenotypes to *KRP2*-overexpressing plants, such as severely dwarfed plants, with enlarged cells, but this increase in cell size was associated with high endoreduplication [71]. *SIM* has an important role in trichome differentiation, being required to suppress mitosis as cells undergo endoreduplication, as sim mutants developed multicellular trichomes, while in *SIM*-overexpressing plants, an extra round of endoreduplication occurred in trichomes and in pavement cells [62,71].

### 3.2. CDK-Activating Kinases (CAK)

Whereas the activity of CDKs can be down-regulated by CDK inhibitors, directly or indirectly, in response to DNA damage, the CDK-activating kinases (CAKs) up-regulate CDKs by phosphorylation of a conserved threonine residue in the T-loop region [72]. Plants have two types of CAKs: CDKD and CDKF. Arabidopsis has three CDKDs (CDKD;1–CDKD;3), where CDKD;2 and CDKD;3 still display kinase activities towards not only CDKs but also the carboxy-terminal domain (CTD) of the largest subunit of RNA polymerase II. When the CDKA/CYCD complex is formed and activated by CAKs, the main phosphorylation target is the retinoblastoma-related protein (RBR), thus activating the E2F/DP complex allowing G1/S transition [73,74].

During meiosis, it was seen that the organization of microtubules is controlled by CDKA depending on activation by CDKD [75]. In order to have a complete CDKA;1 activity, the function of CDKD;1, CDKD;2 and CDKD;3 as CDK activators (CAKs) is necessary [75]. Triple knockout for the CDKD gene was not viable, whereas double mutants cdkd;1 cdkd;2 and cdkd;2 cdkd;3 had lower growth and fertility phenotype; however, double mutant cdkd;1 cdkd;3 showed gametophytic lethality [76,77]. Additional studies found that cdkd;2-1 and cdkd;2-2 mutants showed an acceleration of flowering [33]. 

The other known CAK in plants, CDKF, is known to activate CDKD by phosphorylation of the threonine residue of the T-loop [78]. CDKF is unique to plants and does not depend on association with cyclin to become active. Furthermore, in *Euphorbia esula*, CDKF showed autophosphorylation ability [79]. Studies with the *cdkf;1-1* mutants in Arabidopsis exhibited defects in cell division in both the shoots and roots that showed decreased cell number and cell size in epidermis and mesophyll tissues, in addition to endoreduplication defects. Also, *cdkf;1-1* mutants showed reduced meristem size, slower root development, and smaller leaves that exhibited an abnormal wavy and serrated phenotype. These characteristics indicate that CDKF;1 plays multiple roles in the different processes of cell growth, besides its role in the cell cycle [80].

### 3.3. APC/C and Cyclin Degradation

The proteolysis of cyclins is considered a key process for the correct cell cycle progression. The Anaphase-Promoting Complex/Cyclosome (APC/C) executes a ubiquitin ligase (E3) activity and is essential for the regulation of metaphase to anaphase transition and exit from mitosis [15,81]. APC/C triggers ordered the destruction of mitotic regulators such as cyclin A, cyclin B, and many of the mitotic regulatory kinases, as well as securine, an inhibitor of chromosome separation, allowing the sister chromatids to divide into two daughter cells [81,82]. The degradation of these macromolecules occurs via the 26S proteasome, depending on some APC activator proteins, such as CELL DIVISION PROTEIN 20 (CDC20) and CELL CYCLE SWITCH 52 (CCS52) [15,83]. The APC/C is highly conserved among eukaryotes, composed of at least 14 core subunits already identified in Arabidopsis, with catalytic and activator functions, in addition to subunits with positive and negative regulatory properties [15,84,85]. 

The plant APC/C subunits play a role in many different stages of both vegetative and reproductive development, being essential in gametogenesis [83,86]. Arabidopsis plants overexpressing the APC10 core subunit showed accelerated growth by increased cell numbers that were associated with a faster CYCB1;1 degradation [87]. Furthermore, overexpression of UVI4 and UVI-like/OSD1/GIG1, two APC/C inhibitors, stabilized CYCA2;3 and CYCB1;2 in Arabidopsis [88]. SAMBA is an APC/C negative regulator that also interacts with A-type cyclins (CYCA2;2 and CYCA2;3, weakly with CYCA1;1) and promoted plant growth when mutated [89]. CYCA3;4 is targeted for proteasomal degradation by APC/C^CCS52A2^, and plants with increased CYCA3;4 levels showed disorganized formative divisions, with defects in root meristem and leaf cell differentiation [90].

### 3.4. Transcriptional Regulators of CDKs

In plants, R1R2R3-type MYB (MYB3R) transcription factors regulate the expression of G2/M-specific genes, such as CYCB1, CYCB2, and CDKB2, by binding to the cis-acting element mitosis-specific activator (MSA) present in the promoter region of these genes, thereby controlling the progression of the cell cycle into mitosis and cytokinesis [91,92,93]. Arabidopsis has five MYB3R genes, MYB3R1 to MYB3R5 [94,95,96], where MYB3R1 and MYB3R4 act as transcriptional activators and hence are known as activator-type (Act-MYBs), while MYB3R1, MYB3R3, and MYB3R5 are transcriptional repressors, known as the repressor-type (Rep-MYBs). MYB3R1 has redundant functions acting both as an activator, with MYB3R4, and as a repressor with MYB3R3/5 [91,96,97]. Act-MYBs are phosphorylated and activated by CDKs during G2/M transition, thereby inducing the expression of G2/M-specific genes, including mitotic cyclins, and increasing mitotic CDK activities before the onset of mitosis [96,98]. Rep-MYBs repress the expression of G2/M-specific genes in post-mitotic cells and along the cell cycle, except the G2/M phase [97]. The repressor function of MYB3R3/5 was shown in *myb3r3/5* double mutants by the upregulation of CYCB1;1, CYCB1;2, CDC20.1, and also genes of microtubule-associated proteins with cytokinetic functions, PLEIADE (PLE)/MAP65-3 and ENDOSPERM DEFECTIVE1 (EDE1) [97,99,100]. Moreover, *myb3r3/5* mutants showed an increased organ size, such as leaves, roots, and seeds, and increased cell proliferation in leaves and roots, indicating a role for Rep-MYBs as negative regulators of organ growth mainly by inhibiting cell proliferation [97]. On the other hand, the *myb3r1/4* double mutant was reported to have several defects in morphology, semi-dwarf phenotype, and aberrant cytokinesis, suggesting that these two proteins positively regulate mitosis and are essential for correct development [91,96].

## 4. Regulation Mechanisms of the Cell Cycle and CDK Activity in Environmental Response Signaling Cascades

Plant growth and cell division are often negatively affected in response to various environmental setbacks. The initial perception of stresses responses [101] is followed by intracellular signaling cascades that frequently end with cell cycle delay or cell cycle arrest at G1/S and G2/M by mechanisms that involve inhibition of CDK activity [102]. In the following section, we present data on the regulation of CDKs by intracellular signaling cascades common to various stresses, revealing a role of the cell cycle as a component of environmental response signaling cascades.

### 4.1. DNA Damage Responses and G2 Arrest

Most climate fluctuations that have negative effects on plant growth and productivity induce the generation of Reactive Oxygen Species (ROS) in plant cells, which can cause DNA damage [103]. Various types of DNA damage can be triggered by different stresses, including heat, cold, drought, salinity, UVB radiation from high sunlight, as well as aluminum toxicity, organic pollutants, and pathogens [104]. All living organisms must ensure the maintenance of genome integrity for proper development and faithful transmission of the genetic information from one generation to the next since DNA damage has pleiotropic effects on plant development, morphology, and physiology [105]. CDK/cyclin complexes are key members of cell cycle machinery that are targeted for regulation by the signaling cascade in response to DNA damage, driving cells to G2 arrest. 

**Figure 2:**
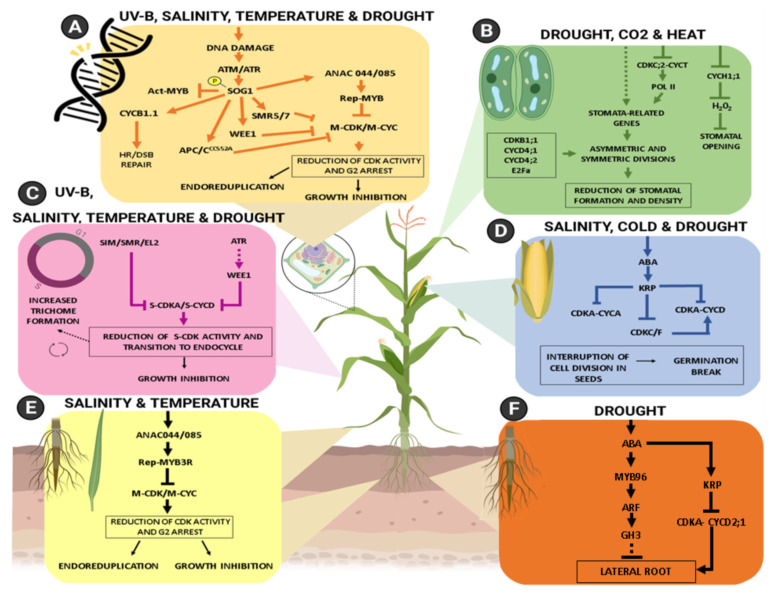
Cyclin-dependent kinases acting as part of intracellular signaling cascades that respond to a number of environmental stresses. Due to climatic events, adaptability strategies are triggered by plants to overcome stressful conditions. Excess CO_2_ in the atmosphere causes temperature fluctuations, intensifying water scarcity, which can aggravate the levels of salt concentration in the soil. To face these limitations imposed by the environment, plants undergo molecular, physiological, and mainly morphological changes. CDKs, together with cyclins, play an important role in primary responses to cellular processes that aid in plant survival. According to the modulation of the environment, strategies such as the interruption of germination (**D**), G1-S arrest by the action of CDK inhibitors SIM/ SMR/EL2 and WEE1 (**C**), and G2 arrest triggered by a decrease in CDK activity as part of a SOG1-dependent DNA damage response that also involves different CKIs (**A**) lead to growth inhibition and often transition to endoreduplication. The reduction in stomatal density, minimizing water loss (**B**) and SOG1-independent cell cycle arrest at G2 leading to growth inhibition in situations of excessive salinity and temperature (**E**) are also considered plasticity strategies. Eventually, it becomes necessary to drive the metabolism towards the formation of new organs, such as the increase of main roots during drought (**F**) and the formation of trichomes stimulated under different stresses to minimize evaporation and regulate temperature (**C**). Abbreviations: ACT-MYB—activator-type MYB3R; REP-MYB—repressor-type MYB3R; HR—homologous recombination; DSB—double strand break; SOG1—suppressor of gamma response 1; ATM—ataxia telangiectasia mutated; ATR—ATM and RAD3-related; DDR—DNA damage response; CDK—cyclin-dependent kinase; CYC—cyclin; APC/C—anaphase-promoting complex/cyclosome; SIM—siamese; CKI—CDK inhibitor; NAC—domain transcription factors; SMR—siamese-related; POL II—RNA polymerase II; ABA—abscisic acid; KRP—KIP-related protein; ARF—auxin response factor; GH3—gretchen hagen 3. Dotted arrow—indirect mechanism; continuous arrow—induction; block arrow—repression. Created with BioRender.com.

During the process of DNA replication in the S-phase, most lesions that occur in the DNA are sensed and repaired by dedicated machinery [102]. However, DNA damage such as Single-Strand Breaks (SSBs) and Double-Strand Breaks (DSBs) require specific DNA repair pathways such as Non-Homologous End Joining (NHEJ) or Homologous Recombination (HR) [106]. In that case, cell cycle checkpoints are activated in a DNA Damage Response (DDR) process that triggers cell cycle arrest to ensure proper DNA repair, or, depending on the severity of DNA lesions, leads to endoreduplication or even Programmed Cell Death (PCD) [107]. The DDR signaling cascade is conserved between plants and animals, and its activation relies on two serine/threonine phosphatidylinositol-3-OH kinase-like kinases, called ATAXIA TELANGIECTASIA MUTATED (ATM) and ATM and RAD3-RELATED (ATR), which are key components of DNA damage response cascades [108]. ATM is primarily activated by DSBs, whereas ATR is activated by SSBs and replicative stress that hampers the progression of the DNA replication fork [109,110] (Figure 2A). In mammals, ATM and ATR phosphorylate and activate CHECKPOINT KINASE 1 (CHK1) and CHK2, which in turn phosphorylate and activate the tumor suppressor protein p53, a transcription factor that controls the DNA damage response [108]. Plants lack functional orthologs of CHK1/2 and p53. Instead, the function of p53 is played by the plant-specific NAC-domain transcription factor SUPPRESSOR OF GAMMA RESPONSE 1 (SOG1), which is directly phosphorylated and activated by ATM and/or ATR. SOG1 induces the expression of hundreds of genes involved in cell cycle regulation, including CDK inhibitors (CKIs), to trigger G2 arrest and allow DNA repair before the onset of mitosis [111,112,113] (Figure 2A).

In Arabidopsis, SOG1 directly upregulates the expression of the CKIs *INHIBITOR OF CYCLIN-DEPENDENT KINASE (ICK)/KIP-RELATED PROTEIN 6 (KRP6)*, *SIAMESE-RELATED 4* (*SMR4*), *SMR5*, and *SMR7* [113,114], and indirectly represses the expression of the Act-MYB MYB3R4 to decrease mitotic CDK activities [115] (Figure 2A). SMR4, SMR5, and SMR7, which interact with CDKA;1 [114,116], are induced by different types of DNA stress [61,114], and transcriptional activation of *SMR5* and *SMR7* by DNA damage depends on SOG1 phosphorylation by ATM [108,114]. However, transcriptional activation of CDK inhibitors was not sufficient to arrest the cell cycle at G2 in response to DNA damage, and Rep-MYBs MYB3R1, MYB3R3, and MYB3R5, which suppress the expression of G2/M-specific genes, were also required for inhibition of cell division during DDR [112,115]. Under normal growth conditions, the accumulation of Rep-MYB proteins was restricted to the S phase, while, under DNA stress, their accumulation was extended to the G2 phase to repress G2/M progression [115]. Rep-MYBs were phosphorylated by A-type and B-type CDKs, and this phosphorylation promoted their proteasomal degradation. Thus, reduction of CDK activity due to transcriptional induction of CDK inhibitors by SOG1 might contribute to G2 accumulation of Rep-MYBs in response to DNA damage [115]. However, the NAC-domain transcription factors ANAC044 and ANAC085, the two closest relatives to SOG1 and also direct transcriptional targets of SOG1 [113], were reported to promote Rep-MYB stabilization in response to DNA damage [117]. ANAC044 and ANAC085 were required for SOG1-dependent cell cycle arrest through Rep-MYB accumulation at G2, but not activation of SMR genes or DNA repair genes [117] (Figure 2A). In the absence of ANAC044 or ANAC085, Rep-MYB did not accumulate and could not inhibit G2/M transition, while the plants exhibited tolerance to DNA damage.

The WEE1 kinase is a negative regulator of CDK activity in different organisms and participates in the replicative stress response in an ATR-dependent way, controlling G2 arrest [118]. Upon replicative stress, ATR induced WEE1 expression probably through SOG1 [113] (Figure 2A). Although CDKA;1 was directly phosphorylated by WEE1 [118], CDKA;1, containing non-phosphorylatable substitutions for Thr14 and Tyr15, could fully complement the cdka;1 mutant both under normal growth conditions and replication stress [119]. Thus, it was proposed that WEE1, differently from its animal counterpart, inhibits cell cycle progression independently of CDKA;1 phosphorylation by phosphorylating other substrates. It was recently found that WEE1 might indirectly inhibit CDK activity during DDR by phosphorylating the E3 ubiquitin ligase F-BOX LIKE 17 (FBL17) and promoting its degradation [120]. FBL17 targets the CDK inhibitors KRP6 and KRP7 for proteasomal degradation [121,122]. Thus, degradation of FBL17 during replicative stress leads to increased stability of these CKIs, contributing to cell cycle arrest [120]. 

While the DNA damage response requires inhibition of mitotic CDK activity to stop progression into mitosis, it was found that, counter-intuitively, CDKB1-CYCB1 complexes are specifically activated to mediate DNA repair through HR, acting as major regulators of DDR in plants [51,123]. Consistent with a role in HR-mediated DNA damage response, *CYCB1;1* was induced in several mutants that suffer from DSBs in an ATM- and ATR-dependent way, being a direct target of SOG1 [51,109] (Figure 2A). Plants lacking CYCB1;1, in combination with one of its related B1-type cyclins, were hypersensitive to intra- and inter-strand DNA crosslinks which require HR repair, and the same response was detected in plants lacking both plant-specific B1-type CDKs (CDKB1s), indicating that CDKB1–CYCB1 activity is required for HR [51]. Accordingly, RADIATION SENSITIVE 51 (RAD51), which is essential for DSB repair through HR, was phosphorylated by the CDKB1–CYCB1 complex in vitro, and its recruitment at damaged loci was found to depend on the presence of CDKB1-CYCB1 activity [51]. These findings establish a dual role for *CYCB1;1*, since, under normal growth conditions, *CYCB1;1* is expressed around the G2/M transition, forming active kinase complexes with CDKB1 and CDKB2 to promote cell division [50,124,125].

### 4.2. ANAC044 and ANAC085: A SOG1-Independent Pathway to Trigger G2 Arrest in Response to Stresses

It appears that arresting the cell cycle at G2 might be a regulatory mechanism employed by plants not only during DDR but also in response to other environmental stresses. While SOG1 was specifically phosphorylated and activated by DNA damage [111], *ANAC044* and *ANAC085* genes were not only induced by DNA damage but also by heat, cold, and salinity stresses [117] (Figure 2F). Upon heat stress, which triggers G2 arrest in maize roots [126], accumulation of Rep-MYB proteins was dependent on ANAC044/ANAC085 but not on SOG1, indicating that heat stress induces *ANAC044* and *ANAC085* through a SOG1-independent pathway [117]. Contrastingly, ANAC044 and ANAC085 were not required for inhibition of G1/S progression triggered by osmotic stress [117]. It was proposed that ANAC044/ANAC085 could function as a hub that senses different stress signals and triggers G2 arrest through stabilization of Rep-MYBs [117]. Indeed, heat, low temperature, and UVB/DNA damage are stresses that trigger G2 arrest in maize and Arabidopsis [117,126,127].

### 4.3. Endoreduplication: An “Adapted” Cell Cycle to Response to Stresses

Different findings suggest that endoreduplication is employed by plants as an adaptive, plastic response to attenuate the effects of stress, although the underlying mechanisms are still being elucidated [128]. After termination of cell division, cells can undergo endoreduplication by driving the endocycle, an alternative form of the cell cycle where DNA replication is repeated without mitosis or cytokinesis, increasing DNA content and ploidy [128]. 

To start the endocycle, mitotic CDK activities need to be reduced to below a level that triggers mitosis [129], and the known interconnected mechanisms that lead to endocycle onset can be affected by environmental factors. One of the mechanisms is transcriptional repression of mitotic CDK and cyclin genes, and, as mentioned above, cell cycle arrest at G2 requires the accumulation of Rep-MYB transcription factors to repress the expression of G2/M-specific genes (Figure 2A,F). The Rep-MYBs were stabilized in response to heat and DNA stress and, under these stresses, roots of *rep-myb* mutants *myb3r3* and *myb3r5* not only showed faster cell division but also later onset of endoreduplication [115]. Interestingly, Rep-MYBs have also been implicated in growth repression under salt stress, but through a different mechanism operating under DNA damage and high temperature, since Rep-MYB transcript and protein levels seem to remain unaltered [130].

A second mechanism that regulates endoreduplication is the proteasomal degradation of mitotic cyclins by the APC/C [129] (Figure 2A). CDKB1;1 is an important negative regulator of endoreduplication [131] that associates with CYCA2;3, a key regulator of ploidy levels [132], to form a functional complex that promotes cell division and suppresses endocycle onset [133]. Degradation of mitotic cyclins by APC/C^CCS52A^, in particular, CYCA2;3, leads to endoreduplication, and this process is accelerated by DNA damage [127,134]. Plants lacking UV-B-INSENSITIVE 4 (UVI4), an inhibitor of the APC/C^CCS52A^ which suppresses endoreduplication through stabilization of CYCA2;3 [88], have a higher DNA ploidy level and resistance to UV-B [135,136]. 

Another important mechanism that contributes to endocycle onset is the suppression of CDK activities by CDK inhibitors [12] (Figure 2A). Several reports have already described that the expression of CDK inhibitor genes *SIM/SMRs* is induced in response to environmental factors. DNA damage induces *SMR4*, *SMR5*, and *SMR7* and inhibits cell division [113,114,137]. Arabidopsis *SMR1/SMR3/SMR5*, rice *EL2*, and maize *ZmSMR4* are induced by heat, cold, drought, salinity, and osmotic stresses, and *SIM*, *SMR1*, *SMR2*, and *ZmSMR4* promote endoreduplication [61,71,114,138] (Figure 2A,C). These findings suggest that SIM/SMRs act as members of environmental response signaling cascades and control cell division and endoreduplication by inhibiting CDK activity. Endoreduplication was involved in mediating plant plasticity during drought responses, when transcription of SMR1 was induced, inhibiting cell division and meristem activity, directly influencing an interruption in the growth of leaves and roots [137] (Figure 2C). The *EL2* gene from rice, which was identified as an inhibitor of CDKA1;1/CYCD, along with SIM, had its mRNA levels induced by cold, drought, and propionic acid, potentially being a gene that links cell cycle progression with plant response to these stresses (Figure 2C) [61].

Endoreduplication is a necessary step in trichome development, requiring SIM for inhibition of CDKA;1/CYCD and transition into the endocycle (Figure 2C), a process that also affects trichome branching and size [62,71,128]. *SIM* mutants developed multicellular trichomes, while in *SIM*-overexpressing plants, an extra round of endoreduplication occurred in trichomes and in pavement cells [62,71]. Trichomes require a few rounds of endoreduplication to achieve full cell size by increasing DNA content since trichomes with compromised endoreduplication often collapse and die [139]. The development of trichomes is controlled by different factors, including the environment, and the morphology and density of trichomes help plants to adapt to different abiotic stresses such as salt, temperature, UV-B, and drought [140](Figure 2C). The presence of trichomes was shown to increase the thickness of the epidermis, and the higher content of long-chain fatty acids in these cells helps to reduce evaporation and regulate temperature [141,142]. Also, trichomes aid plants in absorbing moisture and nutrients from the atmosphere in high-altitude areas [143].

### 4.4. Asymmetric Cell Divisions and Stomatal Remodulation

The disposition of the stomata and their final density are influenced by interactions with the underlying tissues and the environment [144]. Their formation occurs through a series of asymmetric divisions and one symmetric division to form a pair of guard cells (GCs) that are regulated by CDKs and other cell cycle genes. In this process, the meristemoid mother cell (MMC) forms a meristemoid by asymmetric division, which further divides and differentiates into guard mother cells (GMCs), which in turn, divide once symmetrically to produce a pair of GCs. Finally, GC differentiation, pore formation, and GC shape control generate mature stomata [145,146]. Numerous proteins, including kinases, interact directly or indirectly with the cell cycle machinery to regulate stomatal formation [146] (Figure 2B).

Different genetic requirements act during the various stages of stomatal development. Initially, the *TOO MANY MOUTHS (TMM)*, *ERECTA (ER)*, *YODA* and *STOMATAL DENSITY AND DISTRIBUTION 1 (SDD1*) genes are important to guide and control asymmetric divisions [147,148,149,150,151,152]. ER is an important LRR-RLK involved in biotic and abiotic stress responses [153,154,155,156,157]. Homologs to *ER*, *ERL1*, and *ERL2* modulate expression patterns for the correct development of stomata [149,156,158]. Despite their homology, these genes play unique roles in epidermal development. Meristemoid differentiation is consistently inhibited by ERL1 and frequently promoted by ER. However, *ER* also appears to be related to the repression of incoming divisions [149,158]. SDD1 and HIC play important roles in detecting environmental signals for the coordination of stomatal development [150,151,159]. Both are related to the decrease in stomatal density in response to light intensity and CO_2_ concentrations, respectively.

Several regulators of G1/S transition are implicated in the control of cell divisions that form the stomata. *CYCD4* is associated with the formation of stomatal precursor cells in the hypocotyl. Mutants with reduced expression of *CYCD4* showed a reduced number of non-bulging cells, and the inverse was observed in plants overexpressing *CYCD4* [57] (Figure 2B). *CDT1* and *CDC6*, which are normally expressed in stomatal precursor cells, were also involved in stomata formation, as their overexpression led to increased stomatal density [160]. E2Fa overexpression strongly increased the number of asymmetric divisions in the stomatal lineage (Figure 2B). On the other hand, RBR might act as a negative regulator of asymmetric divisions, interacting with the E2F-DP complex [161,162]. Also, FOUR LIPS (FLP) and MYB88 restrict the symmetric cell division of the GMC precursor through their role in the G1/S transition [163]. They bind directly to the CIS region in the *CDKB1;1* and *CDKA;1* promoter suppressing the transcriptional level of these *CDKs* [164] (Figure 2B). In contrast, FAMA is essential to regulate differentiation and proliferation, playing a role in stomatal morphology. Finally, it is suggested that in addition to the transcriptional regulation by FLP and FAMA, cell cycle regulators can also be phosphorylated by kinases acting downstream of YODA or ER [163,164].

## 5. Cell Cycle and Plant Plasticity as Adaptive Responses

Besides the well-known role of plant CDKs as key regulators for an accurate cell cycle progression, to guarantee that plants develop with the correct form, these kinases also participate in the modulation of cell divisions to confer plasticity to plant development. That is a key mechanism for plants to better adapt to climate fluctuations. Adaptive changes in response to challenges imposed by the environment are often common to different stress conditions, as presented in Figure 2. Drought, salt, and heat, as well as high CO_2_, are the main abiotic factors affecting agriculture yields [165,166]. Hormone signaling networks and their crosstalk also respond to several environmental stresses [167], directly influencing the cell cycle and its regulators [168]. They play a fundamental role in the adaptation of plants to environmental changes. However, this topic is too broad and will not be covered in this review. In this section, we will briefly discuss how the various signaling cascades in response to these stresses are integrated to confer plant plasticity and adaptive responses.

### 5.1. Growth Inhibition

Growth inhibition is one of the main responses evolutionarily acquired by plants in stressful situations, being a substantial element due to the decrease in agricultural productivity [165]. This developmental feedback is driven by the regulation of CDKs. The pause in cell cycle progression under salt stress is one of the survival mechanisms in *Brachypodium grasses* where there was an accumulation of *CYCB1;1*, *CDKB1*, and *CDKB2* and a reduction in *CYCD4;1* level at salt concentrations of 100 and 200 mM of NaCl, which suggests a blockage of the cell cycle in the G1 phase (Figure 2C). This hypothesis is supported by the induction of *WEE1*, which is one of the CDKA inhibitors [169] (Figure 2C). Other genes involved in cell cycle regulation have already been shown to have their expression levels altered in response to environmental stresses, such as drought. In cotton, *Gh_D12G2017 (CDKF4)* was strongly induced by drought and salinity, indicating that this may be one of the main molecular regulators of the response to these stresses [170].

Under drought conditions, rice plants also showed a reduction in their growth with decreased height and shoot dry mass and a smaller number of leaves [171]. Under salt and water stress, the levels of *CYCB1*, *CYCA1*, and *CDKA/CDKB* in maize are drastically reduced [172], indicating an interruption in the cell cycle checking process performed by these two groups of cyclins and by the association with CDKA and CDKB [173] (Figure 2A).

The effect of salt stress initially affects young leaves, reducing their growth, and later, with the perpetuation of this condition, the senescence of the plant caused by the accumulation of Na^+^ in the tissues [174,175]. Under this condition, root cells terminate their division early, resulting in a reduced meristem and a delay in root elongation [176] (Figure 2E). Exposed to salt stress, the level of *CDKs*, *CYCA2;1*, and *CYCB1;1* decreased, inhibiting root growth in Arabidopsis [177]. In moderate stress by NaCl, *ANAC044* was up-regulated, and *CYCB1* and *CYCB2* were repressed by Rep-MYB, blocking the cycle at G2/M, which inhibited root growth in Arabidopsis, possibly by a mechanism independent of DDR [130] (Figure 2E).

In Arabidopsis, DNA damage led to CDKB2 degradation [127], while in rice, *OsCDKB2;1* was not affected, suggesting that this CDK acts differently in distinct species [178]. SOG1 promoted G2/M phase arrest in Arabidopsis when subjected to salt stress that triggers DNA damage. It down-regulated *CDKB1;1*, *CDKB2;1*, and *CYCB1;1*(Figure 2A). In contrast, it positively regulated *WEE1*, *CCS52a*, and *E2Fa*, which are involved in the activation of key regulators for endoreduplication, thus favoring the pause in vegetative proliferation and preventing the accumulation of ROS that can lead to serious DNA damage and reduced productivity [179] (Figure 2A).

Seeds are essential for propagation in agricultural production. Different mechanisms such as asymmetric division, mitotic cell division, and endoreduplication occur sequentially to generate a healthy embryo and proper seed germination [180]. Seeds perceive environmental stimuli and control their dormancy through ABA, inhibiting germination or stopping the initial stages of seedling development until more favorable environmental conditions ensue [181,182]. Different stresses induce ABA responses, which in turn stimulate the action of KRPs by stopping the cell cycle and seed embryogenesis in unfavorable situations such as excess salinity in the soil, water shortage, and cold (Figure 2D). KRP-type CKIs have specific roles in embryogenesis and are involved in the ABA-dependent drought stress signaling pathway [183]. KRP1 and KRP2 act to inhibit CYCA and CYCD through CDKA, but not CYCB [184]. Furthermore, yeast two-hybrid assays identified possible targets for KRP, CDKC;2, and CDKF;3, being able to interact with them and regulate the cell cycle and endoreduplication through CDKC/F targets [185] (Figure 2D). Collins et al. [186] showed that CYCDs presented specific expression patterns in the seed, as mutants with ectopic expression of *cycd3;1* retarded embryonic development.

### 5.2. Stomatal Plasticity

Environmental stresses such as elevated CO_2_, high light, climate warming, and water deficit impact the stomatal density, disposition, and opening. The stomata have di-versified evolutionarily as a way to adapt to the environmental scenario and the needs of each species [187]. These adaptive responses include the activity of members of CDKs and cyclins families.

Elevated CO_2_ is the main factor for the greenhouse effect and warmer average global temperatures, thus providing a readjustment in its assimilation by plants [188]. In mung beans (*Vigna radiata*), the exacerbated levels of CO_2_ allowed an increase in biomass as a consequence of increased photosynthetic performance through increased carbon capture [189]. On the other hand, both stomatal conductance and density decrease in this condition [189], although this aspect depends on other environmental factors such as water and light deficiency and may vary by species [190,191]. In Arabidopsis, warm temperatures also reduced stomatal density, probably to decrease water loss during transpiration due to higher temperature [192,193].

As stomata play a fundamental role in regulating the use of water in plants, in situations of water stress, one of the main adaptations is the decrease in the number of stomata [194]. CDKC;2 showed that in addition to affecting the transcription of cell cycle genes, it also affects the transcription of genes involved in the development of stomata. *cdkc;2* plants showed delayed growth, altered leaf shape, and a low stomatal density, which in turn caused the plants to have a low water loss, improving cell turbidity and helping plants to get through the drought [5](Figure 2B).

Cyclin H;1 also had its role related to drought response. *cych;1*; RNAi mutants exhibited a drought tolerance phenotype. The authors proposed that cyclin H;1 regulates the stomatal opening mediated by blue light, controlling the homeostasis of reactive oxygen species, leading the plant to tolerate drought [195](Figure 2B).

Although not well-elucidated, it was shown that a lower stomatal density could improve the efficiency of water use, which explains the reason for the increased photosynthetic potential in plants subjected to high concentrations of CO_2_, since this condition promotes the closure of guard cells and, consequently, a decrease in stomatal conductance, as well as in stomatal density [196].

### 5.3. Root Plasticity

Arabidopsis and several crop species exhibit root plasticity in response to a variety of stresses, such as nutrient limitation, drought, salinity, flooding, and extreme temperatures. The adaptive responses modulate root growth and development, as well as the architecture of the root system, by regulators of cell division, including CDK and cyclins [197].

Responsible for the plant’s nutritional and water supply, the roots are quickly affected by changes that occur in the soil [198]. Chemical signals are sent throughout the plant so that immediate responses are triggered, changing the plant’s morphology in order to reduce water loss and consequently protect it against stress [199,200]. Opposite to what happens in the aerial part, rice plants under drought conditions show an increase in the length of the main root [201], in the number of young roots that provide greater water uptake in the soil (Figure 2F) [202], and lengthening of root hairs, to enlarge the surface in contact with the soil [202,203], different to what is commonly observed in salinity conditions (Figure 2E). Hypoxia, arising during flooding that leads to submersion of the plant, triggers root growth disruption due to a limited oxygen supply to the root cells [204]. As an adaptation to increase the oxygen supply to the root tissue, plants induce the formation of aerenchymatous tissue [205]. New adventitious roots, better adapted to low oxygen, can also emerge from stem nodes [204], a process that involves the activation of cell division in the internode intercalary meristem and in the apical adventitious root meristem [206]. In most plants, the root elongation rate is positively affected by the increase in temperature until a limit is reached, and the root growth rate declines as the temperature continues to increase [207,208]. In contrast, lateral roots show a wide range of different heat responses across species [207].

Root growth is controlled by intrinsic signals. ABA participates in auxin modulation in the root [209], providing a greater secretion of protons at the root tip, maintaining root development and elongation, providing an adaptation to water stress [210]. Added to the accumulation of ABA and consequently a greater transport of auxin at the root apex in moderate drought [210], there is strong evidence that this proliferation mechanism is maintained during water stress, allowing the larger main root to reach deeper levels in the soil [211]. Unlike what happens with the main root, there is a decrease in the number but not in the density of the lateral roots in a situation of water stress [212]. Studies show that lateral root formation is also controlled by auxin [213,214] and ABA [215,216] that induce pericycle founder cells to enter the G1/S phase [173]. Experiments with Arabidopsis using mannitol as a simulation of osmotic stress and soil water deficit showed inhibition in lateral root development, highlighting an influence on drought tolerance [217]. Under drought conditions, these signaling pathways are induced by ABA, causing a response of reduction in lateral root elongation orchestrated by MYB96 (Figure 2F), which induces *AUXIN RESPONSE FACTOR (ARF)*, which in turn stimulates the action of *GRETCHEN HAGEN 3 (GH3)*, inhibiting lateral root formation [199,218,219]. Auxin and ABA act antagonistically, while auxin inhibits KRP, thus allowing pericycle cells to enter the G1/S phase; ABA induces *KRP* expression and inhibits *CYCD2;1*, favoring a marked reduction in lateral roots [69,220] (Figure 2F).

## 6. Conclusions and Future Perspectives

Climate change is a subject of countless studies, and projections predict dramatic consequences to agriculture’s productivity worldwide. Drought, salinity, and extreme temperatures, for example, are setbacks that cause great loss to agriculture, so understanding the molecular mechanisms of plant responses to these situations becomes a powerful tool in the search for adapted and productive genotypes. In this review, we briefly summarized the state of the art of the role of cyclin-dependent kinases, and some of their regulators, in modulating the cell cycle in the face of climate fluctuations. CDK–cyclins have been extensively studied as key regulators of the basic cell cycle machinery, having a role in the accurate rates of cell division to ensure that plants develop into their correct form. However, as sessile organisms, plants evolved mechanisms that confer plasticity in their development to better adapt to the environment’s fluctuations. We aimed to draw attention to what we call the dual role of CDKs in this response: these kinases, which directly regulate cell cycle progression, must in parallel sense environmental signals, modulating cell divisions and plant plasticity to better adapt, driving the cycle through climate fluctuations.

As presented here, an increasing number of studies have revealed the connection of cell cycle regulation to environmental signals. However, the mechanisms involved are not yet fully elucidated, and it is an area that remains to be further investigated. Another exciting point is how these signal transduction pathways that regulate cell divisions meet in the cross-talk between different stresses. Finally, elucidating the molecular responses that plants have evolved to overcome adverse conditions is certainly pivotal for the development of adapted plants that will meet the world’s demand for food in the near future.

## Figures and Tables

**Table 1:** CDK and cyclin mutants with their phenotypes.

Organism	Mutant	Construction	Phenotype	Reference
Tobacco	*cdc2a*	Dominant negative	Reduced histone H1 kinase activity and fewer cells	Hemerly et al., 1995
Arabidopsis	*cdc2a*	Dominant mutant	Affected embryo formation	Hemerly et al., 2000
Arabidopsis	*cdka-1*	T-DNA insert—knockdown	Lethality of the male gametophyte	Iwakawa et al., 2006
	*CDKA;1.N146*	Expressed from the STM-promoter	Defect in cell expansion	Borowska-Wykret et al., 2013
Arabidopsis	*cdka;1*	Destruction box—dead	Reduction of cross-overs	Wijnker et al., 2019
Tomato	*pTPRP-CDKA1*	Overexpression	Fruits with reduced number of seeds and diminished amount of jelly	Czerednik et al., 2015
Maize	*CDKA;1D146N*	Change fromAsp146 to Asn146	Reduced endoreduplication but not cell size	Leiva-Neto et al., 2004
Arabidopsis	*CDKB1;1.N161*	Antisense gene overexpression	Enhanced endoreduplication	Boudolf et al., 2004b
Arabidopsis	*CDKB1;1.N161*	Antisense gene overexpression	Defect in formation of stomata	Boudolf et al., 2004a
Rice	*cdkb1;1*	RNAi	No impact on stomata formation	Qu et al., 2018
Arabidopsis	*cdkb2;1* and *cdkb2;2*	amiRNAs	Meristematic defects	Andersen et al., 2008
Arabidopsis	*cdkd;1 cdkd;3*	T-DNA insert—knockdown	Gametophytic lethality	Hajheidari et al., 2012; Takatsuka et al., 2015
Arabidopsis	*cdkd;1 cdkd;2 cdkd;3*	Knockout	Mutant is not viable	Hajheidari et al., 2012
Arabidopsis	*cdkd;2-1* and *cdkd;2-2*	T-DNA insert—knockdown	Early Flowering	Lu et al., 2017
Arabidopsis	*cdkg;2*	T-DNA insert—knockout	Abnormalities during organogenesis	Zabicki et al., 2013
Rice	*cyca2;1*	RNAi	Reduced stomatal production	Qu et al., 2018
Arabidopsis	*CDKB1;1/CYCA2;3*	Overexpression	Increased endoreduplication	Boudolf et al., 2009
Tobacco	*CYCA3;2*	Antisense expression	Callus and embryo malformation	Yu et al., 2003
Arabidopsis	*CYCA1;2/TAM*	Overexpression	Increased nucleus size in trichomes and guard cell	Jha et al., 2014
Arabidopsis	*cyca1;2/tam*	T-DNA insert—knockdown	Problem in initiating meiosis II; increased nucleus size in trichomes and guard cell	d’Erfurth et al., 2010; Jha et al., 2014
Arabidopsis	*cyca2;134* and *cyca2;234*	T-DNA insert—knockdown	Reduced root length and lateral root formation	Vanneste et al., 2011
Arabidopsis	*cycb1;1*, *cycb1;2*, *cycb1;3*, *cycb1;4*, *cycb1;1/1;2*, *cycb1;1/1;3*, *cycb1;1/1;4*, *cycb1;2/1;4*, *cycb1;3/1;4*	Knockout	No altered root phenotype, but had hypersensitivity to cisplatin	Weimer et al., 2016
Rice	*CYCB2;2*	Overexpression	Increased root growth	Lee et al., 2003
Tobacco	*AtCYCD2*	Overexpression	Acceleration in development	Cockcroft et al., 2000
Arabidopsis	*CYCD3;1*	Overexpression	Increased endoreduplication: switch from cell proliferation to cell differentiation	Dewitte et al., 2003
Arabidopsis	*cyd3*	Knockout	Hyperproliferation of cells in leaves, inability to initiate shoots, and reduction in the cross-sectional area of the vascular cambium	Dewitte et al., 2007; Randall et al., 2015
Arabidopsis	*cyd3 ant-9*	Double mutant knockout	Reduction in the cross-sectional area of the vascular cambium	Randall et al., 2015
Arabidopsis	*CYCD4*	Overexpression	Increase of non-bulging cells	Kono et al., 2007
Arabidopsis	*cycd4*	T-DNA insert—knockdown	Reduction of non-bulging cells	Kono et al., 2007
Arabidopsis	*cdkf;1-1*	Knockout	Slower growth and smaller, wavy leaves with abnormal serration; decreased cell number and cell size; endoreduplication defects; reduced meristem size; retarded root development	Takatsuka et al., 2009

**Table 2:** Cyclin-dependent kinases from Arabidopsis and their homologs in animals.

Type of CDK	Cyclin-Binding Motif	Homolog in Animals	Reference
CDKA	PSTAIRE	CDK1/CDK2	Ferreira et al., 1991
CDKB	PPTALRE or PPTTLRE	Plant exclusive	Imajuku et al., 1992
CDKC	PITAIRE	CDK9	Burssens et al., 1998 Newman et al., 1994
CDKD	SPTAIRE	CDK7	Vandepoele et al., 2002
CDKE		CDK8	Wang e Chen, 2004
CDKF		Plant exclusive	Vandepoele et al., 2002
CDKG	PLTSLRE	CDK10/CDK11	Menges et al., 2005
CKL	(V,I,L)(K,R)FMAREI	CDC2-related proteins	Lessard et al., 1999

**Table 3:** Cell-cycle inhibitor mutants.

Organism	CDK Regulator	Construction	Phenotype	Reference
Arabidopsis	*KRP1/ICK1*	Overexpression	Growth inhibition, cell division and endoreduplication inhibition, CDK activity reduction, morphology alterations	Wang et al. 2000
Arabidopsis	*KPR2/ICK2*	Overexpression	Growth inhibition, cell division and endoreduplication inhibition, CDK activity reduction, morphology alterations	Sanz et al. 2011
Arabidopsis	*SIM*	Overexpression	Leaf growth inhibition, mitosis inhibition, CDK activity reduction, increased endoreduplication	Churchman et al. 2006
Arabidopsis		Mutation	Multicellular trichomes due to decrease in endoreduplication, increased leaf area and proliferation	Walker et al. 2000
Arabidopsis	*wee1*	Mutant/T-DNA insertion	Inhibited growth in stress conditions/DNA damage	De Schutter et al. 2007
Arabidopsis	*APC10*	Overexpression	Increased growth	Eloy et al. 2010
Arabidopsis	*samba*	Mutation/T-DNA insertion	Increase in leaves, roots, seeds, cell number and expansion	Eloy et al. 2012
Arabidopsis	*myb3r3* and *myb3r5*	Mutation/T-DNA insertion	Hyperplasia, developmental abnormalities, and irregular cell divisions during embryogenesis	Kobayashi et al. 2015
Arabidopsis	*myb3r1* and *myb3r4*	Mutation/T-DNA insertion	Defective cytokinesis	Haga et al. 2011
